# The DEsire to DIe in Palliative care: Optimization of Management (DEDIPOM) – a study protocol

**DOI:** 10.1186/s12904-018-0279-3

**Published:** 2018-02-20

**Authors:** Kerstin Kremeike, Maren Galushko, Gerrit Frerich, Vanessa Romotzky, Stefanie Hamacher, Gary Rodin, Holger Pfaff, Raymond Voltz

**Affiliations:** 1 Department of Palliative Medicine, Medical Faculty of the University of Cologne, Cologne, Germany; 2 Institute of Medical Statistics and Computational Biology, University of Cologne, Cologne, Germany; 3 Department of Supportive Care, Princess Margaret Cancer Centre, University Health Network, Toronto, Canada; 4 Institute of Medical Science, University of Toronto, Toronto, Canada; 5 Department of Psychiatry, University of Toronto, Toronto, Canada; 6 Institute of Medical Sociology, Health Services Research, and Rehabilitation Science (IMVR), The University of Cologne, Medical Faculty, Cologne, Germany; 7 University of Cologne, Center for Integrated Oncology Cologne / Bonn (CIO), Cologne, Germany; 8 University of Cologne, Center for Health Services Research Cologne (ZVFK), Cologne, Germany; 9 University of Cologne, Clinical Trials Center Cologne (ZKS), Cologne, Germany

**Keywords:** Desire to die, Suicide, Suicidal ideation, Assisted dying, Wish towards hastening death, Relationship, Multi-professional, General and specialized palliative care, Training, Communication

## Abstract

**Background:**

A desire to die (DD) is frequent in palliative care (PC). However, uncertainty remains as to the appropriate therapeutic response. (Proactive) discussion of DD is not usually part of standard care. To support health practitioners' (HPs) reactions to a patient’s DD, a training program has been developed, piloted and evaluated. Within this framework, a first draft of a semi-structured clinical interview schedule with prompts (CISP) has been developed, including recommendations for action to support HPs’ self-confidence. The aim of this study is the further development of the CISP to support routine exploration of death and dying distress and proactive addressing of a DD.

**Methods:**

This observatory, prospective health services study comprises a three step study design: 1. Revision of the CISP and consensus finding based on semi-structured interviews with patients and a Delphi process with (inter-)national experts, patient representatives and relatives; 2. Increasing confidence in HPs through a 2 day-training program using the consented CISP; 3. A formative quantitative evaluation of conversations between HPs and patients (300 palliative patients at three time points) and a qualitative evaluation based on interview triads of patients, relatives and HPs. The evaluation of conversations will include patient-oriented outcomes, including perceived relationships with HPs and death and dying distress. We will also consider aspects of social inequality and gender.

**Discussion:**

The intervention can provide a framework for open discussion of DD and a basis for enhancing a trustful HP-patient relationship in which such difficult topics can be addressed. The benefits of this study will include (a) the creation of the first consented semi-structured approach to identify and address DD and to respond therapeutically, (b) the multi-professional enhancement of confidence in dealing with patients’ DD and an intervention that can flexibly be integrated into other training and education programs and (c) an evaluation of effects of this intervention on patients, relatives and HPs, with attention to social inequality and gender.

**Trial registration:**

The study is registered in the German Clinical Trials Register (DRKS00012988; registration date: 27.9.2017) and in the Health Services Research Database (VfD_DEDIPOM_17_003889; registration date: 14.9.2017).

## Background

A desire to die (DD) is reported by up to half of all terminally ill patients [1–4]. Patients’ expressions concerning the end of their lives can differ in form, intensity and function [5–9]. DD may include an acceptance of death, a desire that the end would come earlier than it would otherwise occur during the course of the illness but without taking any direct action to hasten death, but also includes desires for hastened death, requests for assisted dying, or suicidal ideation/action plans [5, 6, 8, 10]. We use the term “DD” for a broad concept of wanting to die that can occur with a simultaneous will to live [9], has various meanings [10, 11], and many underlying factors [8, 12, 13].

Studies have shown that 12–30% of physicians are confronted with DD [14–16]. Still, DD is not routinely assessed in clinical practice in general and specialist palliative care. Although DD is prominent in palliative care, even health practitioners in specialized palliative care reported feeling uncertain about how to respond to DD [17]. This insecurity can lead to neglecting or insufficiently discussing the issue.

A DD is connected with physical and psychological distress [18]. It can be the beginning of a suicidal process [19], but studies have shown that communication concerning therapeutic options can prevent suicides [20]. Experience from psychiatry shows that starting a conversation on suicide may ease patient’s distress, burden, and may even prevent suicides [21]. This may be done in a manualized way (see Managing Cancer and Living Meaningfully; CALM) [22, 23]. Although the German National Ethics Advisory Board 2014 has suggested suicide prevention programs as countermeasures to assisted suicide [24], psychiatry is rarely involved in palliative care. Proactively addressing DD may be positively perceived by patients [25], opens emotional communication even in absence of DD [2], and contributes to building a trusting relationship, which can help to preserve the will to live [26]. According to a previous study, health practitioners (HPs) perceive establishing and maintaining a helpful relationship as absolutely essential in addressing patients’ DD [27].

Patients can express a DD in different ways. They often affirm their wish to go on living and appreciate their positive experiences, but nevertheless refuse to live under the current conditions [12]. A DD may also be a manifestation of letting go [16]. Ohnsorge et al. [10] differentiated DD into three categories. First, a hope or longing for dying, but without a desire for hastened death. In a second category, a desire for hastened death (DHD) is a hypothetical option, such as in the case of an increase in symptoms. Third, the DD may occur in the way of an explicit desire to be killed, a desire for withdrawal of life-sustaining measures in order to hasten death or in the form of (desire for assisted) suicide. Moreover, patients with DD can simultaneously express a will to live [1, 28].

Although patients’ gender and social inequality distribution in palliative care are important to take into account [29], these variables have not been well explored in regard to DD. It is known that individuals with a low socio-economic status are more likely to be affected by mental disorders such as depression than others [30–32]. Furthermore, a lower socio-economic status is significantly associated with higher risk of suicide [33] and negatively influences patients’ access to health care services, including specialist palliative care services [34, 35]. This may result in a greater likelihood of DD and the need for greater cultural awareness and sensitivity of HPs [36]. In addition, there are gender differences in DD [37] and the will to live [38]; women score higher on the Schedule of Attitudes towards Hastened Death (SAHD) than men, express a significantly weaker will to live, and less desire to prolong life by medical interventions. Some studies report being female as a risk factor for developing a DD in older people [39, 40]. Other studies showed that men have lower rates of attempted suicide compared to women but a higher rate of completed suicides because of their more lethal suicidal behavior [41]. These findings underline that HPs need to be aware of socio-economic aspects and gender bias in regard to DD.

Proactively exploring DD may clarify its underlying motivation at an early stage and improve the HP-patient relationship through such open conversation, thereby reducing distress and possible requests for assisted suicide. A recent study showed that HPs could benefit from a training to further improve skills and confidence with regard to dealing with DD [27]. Therefore a training program has been developed, piloted, and evaluated to support HPs in addressing a patient’s DD [42]. Within this framework, the content of a 2 day-training program and a first draft of a clinical interview schedule with prompts (CISP) to deal with a DD have been developed. The CISP is a semi-structured guideline for highly individual situations and includes recommendations for action in order to ease patient’s suffering. It can help HPs to focus on what is important to address in such conversations (e.g. in order to not avoid it). Since it may be a significant experience of patients with advanced disease at some time [43], exploring death and dying distress and allowing emotional expression can be helpful. A special focus of the CISP is on establishing and maintaining a helpful relationship, as this was found to be essential to address DD [27]. The impact of the CISP on patient-relevant outcomes has not yet been examined.

This study aims to

- further develop and consent the CISP drafted to routinely assess death and dying distress and to proactively address DD.
- strengthen confidence of HPs in dealing with patient’s DD through the evaluated 2 day-training program using the consented CISP. This is intended to reduce HPs uncertainties and deepen the HP-patient relationship so that such difficult topics can be addressed.
- quantitatively and qualitatively evaluate conversations between HPs and patients to test the outcome of the intervention (primarily, improvement of HP-patient relationship and decrease of death and dying distress) in a broad range of clinical situations considering social inequality and gender aspects.

## Methods/ Design

**Fig. 1:**
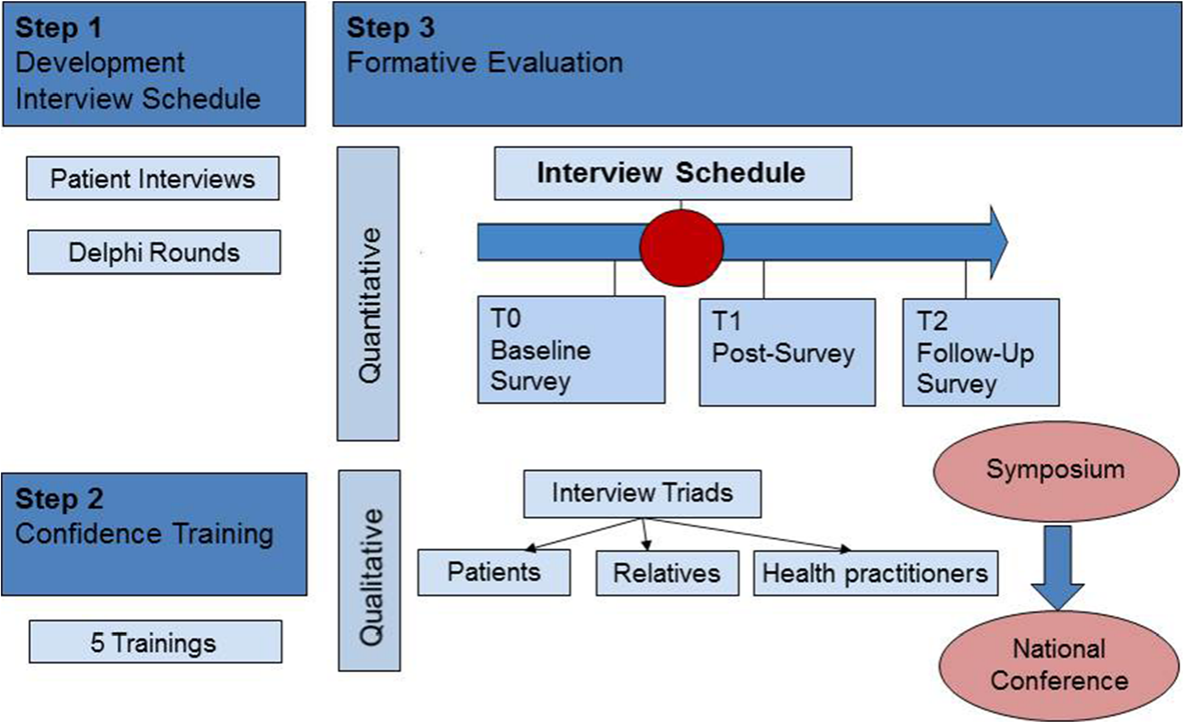
Study design

This health services research study aims at designing and testing an intervention to proactively assess DD and routinely addressing death and dying distress. It consists of three steps (see Fig. 1):

1. The revision of and consensus finding on an existing first draft of a clinical interview schedule with prompts (CISP) to deal with a DD by a) semi-structured interviews with patients, and b) a Delphi process with international experts, patient representatives and relatives.
2. Increasing confidence in HPs through a piloted and evaluated 2 day-training program using the consented CISP. The training course and HPs’ knowledge, skill and self-confidence gain due to the training as well as their personal experience in using the CISP will be evaluated.
3. An evaluation of conversations about possible DD between HPs and patients: quantitatively using a baseline survey followed by two follow-ups with patients; qualitatively by interviews with triads of patients, relatives and HPs (10–15 each).

Step 1: Revision of and consensus finding on the interview schedule to deal with a desire to die.

### Objective /research questions

An existing first draft of the CISP to deal with a DD and its possible benefits for clinical practice shall be discussed, reflected, revised and consented.

### Participants

Participants will be palliative patients, professional experts, patient representatives and relatives.

### Data collection/procedures and sample

#### Semi-structured interviews with patients

Semi-structured interviews with patients will be conducted to strengthen the appropriateness of the CISP. An interview guideline will be used to ensure that all relevant hypothesis-directed and theory-driven topics of the CISP will be covered [44], and that open answers will be obtained in order to gain and reconstruct the patients’ subjective perspective. As palliative patients are often limited in their capacities (e.g. because of their limited ability to concentrate or decreased mobility), one-on-one interviews are most appropriate and feasible (in contrast to e.g. focus groups).

We aim to include a heterogeneous sample of 10–15 patients receiving general or specialist palliative care in different inpatient and outpatient care settings (e.g. palliative, oncological, neurologic/psychiatric and geriatric wards, senior residences, palliative home or outpatient care). For patients’ inclusion and heterogeneity criteria see Table 1.

**Table 1:** Criteria for inclusion and heterogeneity in patient interviews

Inclusion criteria	Heterogeneity criteria
a) Adult palliative patients b) Conditional and cognitive ability to participate in an interview c) Adequate German language skills d) Willingness to participate and presence of written declaration of consent	a) Care setting b) Diagnosis c) Age d) Gender e) Socio-economic status f) Cultural background

Interviews will take place at the desired location of the patient, e.g. the care setting. The interviews will be recorded and have an estimated duration of 30 to 60 min. Interviewees are free to take a break or interrupt the interview at any time. Even though we have found in previous studies that patients appreciate conversations on death and dying and do not typically experience discussion of DD as burdensome [28, 45], we will observe whether talking about these existential issues triggers emotional distress and/or the need to modify the conversation or to provide support. To this end, we will check in with the patients the day following the interview if possible (or the next possible time the patients are available) to ask how they feel and if there is anything they want to talk about. The patients health care teams will also be informed that the interviews are being conducted and optional psychological support for the interviewer and the interviewees in case of need will be ensured.

#### Delphi procedure with professional experts, patient representatives and relatives

In addition to the adaption of the CISP based on the patient interviews, a Delphi procedure will be conducted to consent content and structure of the CISP. The Delphi technique is used for the formation of consensus on existing knowledge and the current conceptual world [46, 47]. For our Delphi we will apply the criteria presented in Table 2 for selection of 50–70 (inter-) national experts after taking “panel mortality” into account. To include palliative patients themselves would of course be valuable but we cannot expect severely ill patients with typically short life expectancy [48] to take part in a Delphi process with several rounds of inquiry over a period of time.

**Table 2:** Criteria for including professional experts in the Delphi process

Inclusion criteria
a) Occupational group (multi-professional plus ethical experts, patient representatives)
• Experience with DD and/or suicidality
• High scores in self-assed confidence and expertise in DD
b) Patient representatives
c) Relatives

An online tool will be used for the iterative process of administering two to three rounds of surveys to the Delphi panel. Panel members will respond to statements using rating scales to express their agreement or disagreement regarding the relevance of several aspects in dealing with DD. Text boxes for panel comments will be provided. A priori criterion for consensus will be defined [49]. All material provided to the expert panel at the outset of the project and throughout the Delphi process will be carefully reviewed and piloted in advance in order to examine the effect on experts’ judgements.

### Data analysis

The interviews will be transcribed verbatim and analyzed according to qualitative content analysis [50–52]. Inductive and deductive categories will be derived and applied with the aid of the qualitative data analysis software MAXQDA 12.

Quantitative statistical analysis of the experts’ ratings will be undertaken using SPSS Statistics 22 (or higher) (IBM Corp., Armonk, NY, USA). Thresholds and definitions of consensus will be based on values used in previous studies [53–56].

Step 2: Increasing confidence in health practitioners by a 2 day-training program using the consented clinical interview schedule (CISP) developed in step 1.

### Objective /research questions

Following the revision of and consensus finding on the CISP, multi-professional training courses will be conducted using the consented CISP to increase HPs’ self-confidence in dealing with DD.

### Participants

Participants will be multi-professional HPs in specialist but also general palliative care from various disciplines (oncology, neurology, geriatrics, palliative care). This broad approach takes into account that most palliative care patients are cared for in general palliative care settings rather than in specialized settings [57].

### Procedure and sample

In implementing the training courses, the results of our preliminary work can be referred to (Frerich G*, Romotzky V*, Galushko M, Perrar KM, Montag T, Doll A, Kremeike K, Golla H, Strupp J, Voltz R. How to deal with a patient´s desire for hastened death: Development of a teaching course for health professionals in palliative care, submitted; Romotzky V*, Frerich G*, Galushko M, Hamacher S, Kremeike K, Voltz R. Let’s talk about the desire to die - evaluation of a training course for health professionals, in preparation). Within a previous project, the training concept was developed, piloted and evaluated. Based on the evaluation, the curriculum was revised. The CISP will be applied continuously during the training.

Five training courses will be conducted, each with 12 health care professionals from various professions and disciplines as well as hospice staff members. HPs will be purposefully sampled; eligibility criteria for participating in the course are presented in Table 3.

**Table 3:** Criteria for course participation

Participation Criteria
a) Belonging to one of the multi-professional occupational groups in palliative care (e.g. physicians, nurses, social workers, psychologists, hospice staff members)
b) Involvement in direct in- or outpatient specialist or general palliative care for at least 3 years
c) Adequate German language skills

Course participants will be recruited from the Cologne region. This geographical limitation allows an evaluation of the training impact by personal face to face interviews with HPs, patients, and relatives in step 3. Taking part in the training course is voluntary and free of charge for HPs. Following the courses, participants will be asked to use the CISP in addition to the competences learned in the training course, when discussing DD in routine consultations. By participating in the training course, HPs explicitly agree to recruit patients they will discuss a DD with to be enrolled in step 3 of this study.

**Fig. 2:**
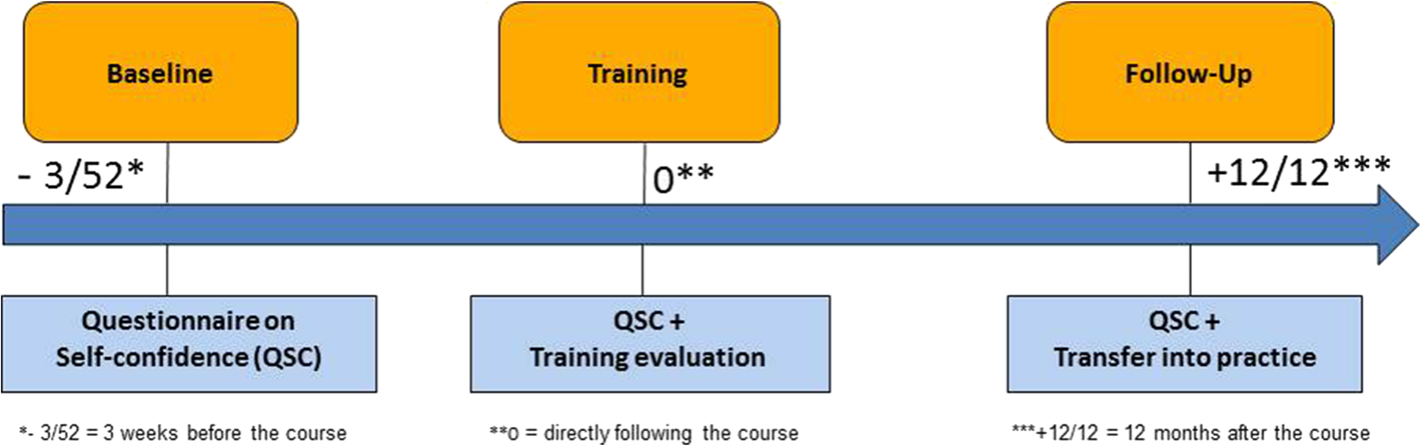
Training course evaluation

Training courses will be evaluated based on changes in the HPs’ self-confidence in dealing with DD as well as their knowledge, skills and attitudes related to the topic. Self-confidence will be assessed at three measurement times (3 weeks before the course, directly following the course and 12 months after the course; see Fig. 2). At the third time point, additional follow-up questions will be asked regarding practical applicability of the learned competences and the use of the CISP. To this end, a standardized questionnaire developed in the preliminary project will be used [42]. Additionally, health professionals will be asked to reflect upon their consultations with patients with DD taking part in step 3 of this study. This reflection will also include an inquiry of the specific content discussed with patients and potential burden for the HPs and patients while talking about DD. Due to the HPs’ training, an increase of self-confidence in HPs for such conversations is expected, which will be measured using the confidence scale developed in the course of the preliminary project.

Step 3: Formative evaluation of conversations on desire to die between health practitioners and patients.

### Objective /research questions

Qualitative and quantitative evaluation of conversations on DD between patients and HPs using the CISP developed in step 1 and the training course content provided in step 2.

### Participants

Participants will be palliative patients, their relatives and HPs from the Cologne region.

### Procedure and sample

As combining qualitative and quantitative methods provides a more complete view on the research topic than including only one type of data [58, 59], we will use semi-structured interviews as well as standardized and validated questionnaires to evaluate conversations on DD between HPs and palliative care patients. Given how the intervention is still under development, this study is prospective and observatory. The study will investigate whether there is no harm caused by the intervention and will set the ground for a future randomized controlled trial (RCT).

Criteria for patient inclusion and sample heterogeneity are similar to those applied in step 1 (see Table 1) supplemented by one inclusion criterion: a life expectancy of 3 to 12 months (surprise question). Each of the 60 HPs trained in step 2 will be asked to recruit about 5 patients for the evaluation of conversations on DD. A total of 300 patients for quantitative analysis will enable us to detect even small within-group effects (with α = 5% and 1 – β = 80%, i.e. < 0.2 Cohen’s d for the whole group; in subgroups of *n* = 40 < 0.5 “moderate”). Patient sampling will be consecutive including every eligible patient fulfilling the inclusion criteria from different care settings (inpatient and outpatient, palliative, oncological, neurologic/psychiatric and geriatric wards, senior residences).

### Data collection

#### Quantitative

As patient relevant outcomes of an open conversation about possible DD we consider primarily the improvement of relationship-quality and the decrease of death and dying distress. These outcomes will be measured by the Patient-Doctor-Relationship Questionnaire German Version (PDRQ-9) [60, 61] and the Death and Dying Distress Scale (DADDS) [62–64]. The PRDQ-9 was developed as a short assessment of the relationship between primary care physicians and patients from the patient’s perspective (9 items on a five-point Likert-scale) and is validated in German [60, 61]. The DADDS assesses specific concerns of advanced cancer patients with regard to insecurity about ones end of life, being a burden to others, as well as lost time and opportunities [62–64]. The German adaptation includes 9 instead of 15 items [65]. Additionally, the Likert-scale was shortened from a six-point to a five-point Likert-scale with mild and moderate distress put together to one label.

As secondary outcomes, DHD and suicidal thoughts are expected to decrease. A DHD as one aspect of DD can be measured by the SAHD [6, 66], which was also validated in German with patients in specialized palliative care units [6, 66]. The clinical course of patients’ depression (Patient Health Questionnaire; PHQ9) [67–69]; hopelessness (Beck Hopelessness Scale) [70] and the will to live (Visual Analogue Scale; VAS) [71, 72] will also be documented. Table 4 gives an overview about the instruments that will be used for the quantitative evaluation with patients.

**Table 4:** Instruments for the quantitative patient survey to evaluate conversations between palliative care patients and health practitioners

Instrument	Use	N Items, Answering Options	Estimated completion time	Validation/ Population
PDRQ-9 - Patient-Doctor-Relationship Questionnaire German Version	Assessment of patients’ perceived therapeutic alliance with primary care physicians	9, 5 point Likert scale (from 1 = not correct at all to 5 = fully correct)	5 min	Validated in German/ General population
DADDS - Death and Dying Distress Scale German Version	Assessment of patients‘death anxiety	9, 5 point Likert scale (from 0 = not distressed by this thought or concern to 4 = extreme distress)	5 min	- / Patients with advanced or metastatic cancer
SAHD-D – Schedule of Attitudes Towards Hastened Death German Version	Assessment of patients‘desire to hasten death	20, yes/no (true/false)	10 min	Validated in German/ Patients in specialized palliative care units
PHQ9- Patient Health Questionnaire	Facilitate detection of depression according to DSM-IV criteria	9, 4 point Likert scale (0 = Not at all, 1 = Several days, 2 = More than half the days, 3 = Nearly every day)	5 min	Validated in German/ Patients in primary care
BHS - Beck Hopelessness Scale	Assessment of hopelessness and depression	20, true/false	10 min	Validated in German
VAS - Visual Analogue Scale	Assessment of will to live	1, 100 mm-sacle from complete will to no will	Completed in seconds	–
Total		74	30–40 min	

**Fig. 3:**
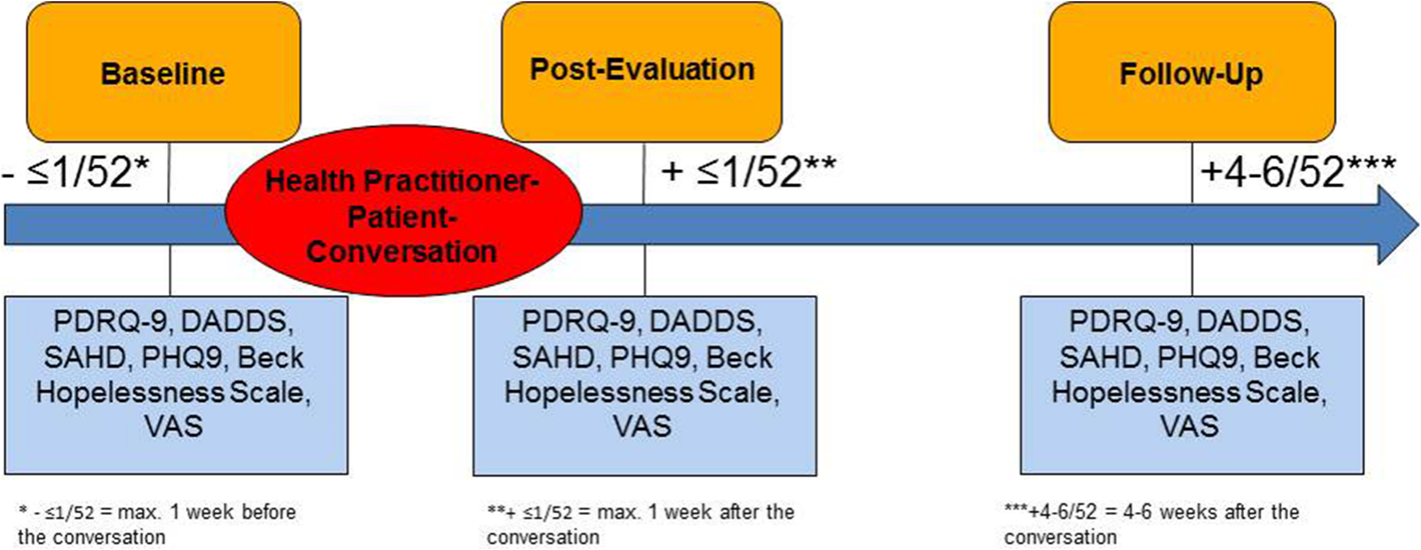
Timeline of the quantitative evaluation of conversations on DD between palliative care patients and health practitioners

To complete the aforementioned questionnaires, the research team will conduct personal quantitative interviews with each patient at three measurement times (1. max. 1 week before the HP-patient-conversation on DD, 2. max. 1 week following the conversation, and 3. four to 6 weeks after the conversation; see Fig. 3). At the second measurement time, patients will additionally be asked to name the content discussed during the consultation.

After patients have been recruited by the HP to participate in step 3, the research team will inform them about the study. Only after these patients have signed a declaration of consent, interviews will take place at the desired location of the patient, e.g. within the care setting. A copy of the questionnaire’s response categories will be handed out to the interviewees. The interviewer will note the patients’ answers for them. Each quantitative interview will have an estimated duration of 30 to 40 min. Interviewees are free to take a break or interrupt the interview at any time. As the interview might raise emotional disturbance and/or the need for discussing some issues in more detail, we will check in with the patient the day following the interview if possible (if not, the next possible time) to ask how they feel and if there is anything they want to talk about. The patient’s health care team will also be informed that the interview is conducted. At all three measurement times, it will be checked before conducting the inquiry, whether patients are conditionally and cognitively able to participate in an interview.

#### Qualitative

In addition to the quantitative evaluation, semi-structured qualitative interviews [73, 74] with 10–15 sets of patients, their relatives and their HP trained in step 2 will be conducted to gain a deeper insight into their perception of the conversation about DD between patients and HPs [44, 75]. As one of the main outcomes is the quality of the relationship between patients and the HPs, we chose triads to account for interactional aspects. Moreover, this will allow us to obtain three perspectives on the identical care situation [76]. Criterion sampling [77] will be conducted to depict the variance of patients’ characteristics and settings.

After patients have participated in the quantitative survey, 10 to 15 of them, and in each case one of their relatives, will be asked to participate in an additional semi-structured qualitative interview. The request to attend a qualitative interview will be placed by a HP trained in step 2, who is also willing to participate in a qualitative interview. If HP, patients and relatives agree to participate, the research team will inform them about the study. Only after HP, patient and relative all have signed a declaration of consent for the triads individual interviews will be conducted at the desired location of the interviewees, e.g. within the care setting. The interviews will be recorded on tape and will have an estimated duration of 30 to 60 min. Interviewees are free to take a break or interrupt the interview at any time. As the interview might raise emotional disturbance and/or the need for discussing some issues in more detail, we will check in with the HP, patient and relative the day following the interview, if possible (if not the next possible time) to ask how they feel and if there is anything they want to talk about. The patient’s health care team will also be informed that the interviews are being conducted and optional psychological support for the interviewer and the interviewees in case of need will be ensured.

### Data analysis

#### Quantitative

The full quantitative analysis set as derived from the intention-to-treat (ITT) principle includes all patients with a valid assessment at t0 (baseline survey) and t1 (post-survey). The primary outcome “change in PDRQ-9 from t0 to t1” is analyzed by a two-sided paired t-test with a significance level of 5%. The corresponding null hypothesis is “H0: The Patient-Doctor-Relationship (measured with PDRQ-9) does not change from t0 to t1” and the alternative hypothesis “H1: There is a change in Patient-Doctor-Relationship from t0 to t1”. Additional explorative subgroup analyses (ANCOVA) are done by sex, age, socio-economic status, social inequalities and setting. The secondary outcomes and sensitivity analyses for change in PDRQ-9 from t0 to t2 are essentially analyzed along the same lines as the primary outcome, i.e. using paired t-tests. Moreover, single and multiple imputation methods are applied to analyze the impact of competing events (i.e. ADD, ADI, AaR) assuming not-at-random missing-patterns [78, 79]. Adverse events are aggregated by category and listed. Time-to-event (e.g. survival) distributions are summarized by the Kaplan-Meier method and subgroups are compared by the (stratified) log-rank test. For descriptive analyses qualitative data are summarized by count (percentage), quantitative data by mean, standard deviation and percentiles (0, 25, 50, 75, 100). To pay attention to the interrelation of various personal characteristics (intersectional theory) [80], the analysis of results will include testing the correlation of these diverse characteristics including socio-economic and cultural background as well as gender and CISP outcomes.

#### Qualitative

The interviews will be transcribed verbatim and analyzed according to qualitative content analysis [50–52] as described in step 1. In addition, the triangulation of data from patients, their relatives and their HPs facilitates a case-related analysis of all three perspectives and allows to compare and interrelate the different perspectives – also beyond case level [76]. Constant comparison method will be applied to find differences in patients, relatives and HP concerning for example age, gender, socioeconomic status or type of DD.

Results will be discussed in an expert meeting for finalization of the CISP and the corresponding training. Findings of the formative evaluation will be considered in order to better deal with different kinds of DD and in order to disseminate the CISP and corresponding training. If analyses indicate that no harm is inflicted by proactively addressing DD as suggested by the CISP, further research including a RCT could follow.

## Discussion

We propose that proactively addressing a DD can build a solid basis for open discussion and enhancing a trusting HP-patient relationship in which such difficult topics can be addressed. Furthermore, patients can express a wish to die in different ways and proactively addressing DD as well as managing it may clarify reasons more openly and at an earlier stage.

As previous studies have shown, end-of life research with patients is not only feasible, but also welcomed by patients [28, 45]. We do not expect harm when initiating careful discussion on DD with patients. Patients and relatives mostly want to discuss end-of-life issues and often hope this to be initiated by their HPs [81–84]. They also wish for a HP who is comfortable in such discussions [85–87].

The potential benefits of this study are: 1. The international creation of the first semi-standardized approach for a proactive assessment of a DD and the optimal therapeutic response to it, 2. the multi-professional enhancement of confidence in dealing with DD by a training course which can be integrated into other training programs, and 3. the improvement of managing DD for patients, relatives and HPs considering aspects of social inequality and gender.

## Abbreviations

AaR: Attrition at random
ADD: Attrition due to death
ADI: Attrition due to illness
BHS: Beck Hopelessness Scale
DADDS: Death and Dying Distress Scale
DD: Desire to Die
DHD: Desires for hastened death
HP: Health practitioners
PDRQ-9: Patient-Doctor-Relationship-Questionnaire German Version
PHQ9: Patient Health Questionnaire
RCT: Randomized Controlled Trial
SAHD: Schedule of Attitudes towards Hastened Death
SAHD-D: Schedule of Attitudes towards Hastened Death German Version
VAS: Visual Analogue Scale
